# Using DNA affinity purification sequencing (DAP-seq) to identify *in vitro* binding sites of potential *Novosphingobium aromaticivorans* DSM12444 transcription factors

**DOI:** 10.1128/mra.00260-25

**Published:** 2025-07-22

**Authors:** Kevin S. Myers, Alexandra M. Linz, Leo A. Baumgart, Yu Zhang, Ian Blaby, Ritesh Mewalal, Vivian Ng, Daniel R. Noguera, Timothy J. Donohue

**Affiliations:** 1 Wisconsin Energy Institute and Great Lakes Bioenergy Research Center, University of Wisconsin-Madison 5228, Madison, Wisconsin, USA; 2 U.S. Department of Energy, Joint Genome Institute, Lawrence Berkeley National Laboratory, Berkeley, California, USA; 3 Department of Civil and Environmental Engineering, University of Wisconsin-Madison 5228, Madison, Wisconsin, USA; 4 Department of Bacteriology, University of Wisconsin-Madison 5228, Madison, Wisconsin, USA; Loyola University Chicago, Chicago, Illinois, USA

**Keywords:** transcription factor, aromatic metabolism, DAP-seq, bacteria

## Abstract

Genome-wide binding sites of 44 putative transcription factors (TFs) from *Novosphingobium aromaticivorans* DSM12444 were analyzed using DNA affinity purification sequencing. We report that 32 of these TFs have at least one area of enrichment. These data will help better understand aromatic metabolism and other features of *N. aromaticivorans* biology.

## ANNOUNCEMENT

*Novosphingobium aromaticivorans* is an Alphaproteobacterium that can convert aromatic compounds from lignocellulosic biomass into commodity chemicals (1–6). Understanding the transcriptional regulation of the genes encoding proteins that metabolize aromatic compounds would allow for better engineering of *N. aromaticivorans* for the production of commodity chemicals. DNA affinity purification sequencing (DAP-seq) is a method that maps transcription factor (TF) binding genome-wide *in vitro* (7, 8). We selected 44 predicted *N. aromaticivorans* TFs (Table 1) based on their potential association with aromatic metabolism (e.g., upstream of genes that encode proteins involved in aromatic metabolism) or observed changes in transcript abundance during growth on aromatic compounds (1, 3, 9). *N. aromaticivorans* genomic DNA was purified from cultures grown in 2.5 mmol/L vanillic acid and 2.5 mmol/L glucose at 30°C in standard mineral base media (3, 5). The genomic DNA was ultrasonically sheared to an average size of 75 bp, and 1 µg was used to create the sequencing library using the KAPA HyperPrep Kit and standard manufacturer’s protocols (8). Approximately 500 ng of purified linear expression templates were used for *in vitro* protein expression, inclusive of an N-terminus HaloTag (Promega) (8) in a total volume of 50 µL of the TnT coupled transcription/translation reaction (Promega L5540) and incubated at 30°C for 2 hours. The expressed protein was combined with an equal volume of binding/wash buffer (1× PBS, 0.1% Tween-20) containing 10 µL of pre-washed Magne HaloTag beads (Promega G7281), 1 ng of prepared genomic DNA libraries, and 1 µg of salmon sperm DNA. This mixture was incubated at room temperature with gentle rotation for 1 hour and then washed four times with binding/wash buffer. The beads were used directly as templates in a 20 µL PCR (KAPA HiFi HotStart reaction, Roche KK2601) with indexing primers as described previously (8) for 20 cycles to amplify TF-bound DNA fragments. Four negative control samples containing mock protein expression products were included in the DAP-seq assay to produce an average background file that was used for peak calling. The libraries were sequenced on an Illumina NovaSeq 6000 S4 instrument using 2 × 151 bp reads (8). Total read counts ranged from 943,766 reads to 9,167,036 reads with an average read count among all the samples of 2,880,796 reads.

**TABLE 1:** Transcription factors examined by DAP-seq^*a*^

Locus tag	Old locus tag	TF family	Peaks	SRA
SARO_RS09330	Saro_1863	IclR	1,205	SRR32559725
SARO_RS18620	Saro_3549	Helix-turn-helix	375	SRR32559713
SARO_RS19770	Saro_3779	LuxR	246	SRR32559743
SARO_RS17620	Saro_3967	MucR	185	SRR32559728
SARO_RS08540	Saro_1703	TetR	77	SRR32559706
SARO_RS10965	Saro_2178	Helix-turn-helix	67	SRR32559742
SARO_RS15450	Saro_3045	RpoH sigma32	61	SRR32559721
SARO_RS02260	Saro_0451	TetR/AcrR	57	SRR32559740
SARO_RS20390	Saro_3454	MarR	38	SRR32559720
SARO_RS04885	Saro_0978	LysR	35	SRR32559741
SARO_RS09450	Saro_1887	LacI	22	SRR32559708
SARO_RS19415	Saro_3709	TetR/AcrR	19	SRR32559733
SARO_RS04920	Saro_0985	MucR	12	SRR32559722
SARO_RS03775	Saro_0755	LacI family DNA-binding	12	SRR32559729
SARO_RS17250	Saro_3889	IclR	10	SRR32559735
SARO_RS05355	Saro_1073	MarR	9	SRR32559712
SARO_RS07435	Saro_1482	XRE	9	SRR32559737
SARO_RS08650	Saro_1728	LacI family DNA-binding	8	SRR32559734
SARO_RS00810	Saro_0164	GntR	6	SRR32559747
SARO_RS06030	Saro_1206	Unknown	4	SRR32559730
SARO_RS07375	Saro_1471	TetR/AcrR	4	SRR32559738
SARO_RS14285	Saro_2816	LysR	2	SRR32559710
SARO_RS06685	Saro_1336	Hydrogen peroxide-inducible gene activator	2	SRR32559727
SARO_RS12110	Saro_2407	IclR	2	SRR32559732
SARO_RS13730	Saro_2704	Helix-turn-helix	2	SRR32559707
SARO_RS20125	Saro_0784	TetR/AcrR	2	SRR32559746
SARO_RS02605	Saro_0521	TetR/AcrR	1	SRR32559748
SARO_RS02650	Saro_0532	TetR/AcrR	1	SRR32559744
SARO_RS04370	Saro_0873	LysR	1	SRR32559709
SARO_RS07420	Saro_1479	MerR	1	SRR32559718
SARO_RS11485	Saro_2281	Phosphate regulon	1	SRR32559745
SARO_RS17345	Saro_3908	MarR	1	SRR32559731
SARO_RS10140	Saro_2016	CRP/FNR	0	SRR32559714
SARO_RS02445	Saro_0488	CRP/FNR response regulator	0	SRR32559726
SARO_RS00970	Saro_0194	IscR	0	SRR32559705
SARO_RS08575	Saro_1712	TetR/AcrR	0	SRR32559724
SARO_RS00615	Saro_0124	CarD	0	SRR32559749
SARO_RS07800	Saro_1557	IclR	0	SRR32559711
SARO_RS07980	Saro_1592	Sigma70	0	SRR32559715
SARO_RS09350	Saro_1867	AraC	0	SRR32559739
SARO_RS11895	Saro_2364	MerR	0	SRR32559750
SARO_RS13010	Saro_2581	CRP/FNR	0	SRR32559716
SARO_RS14395	Saro_2838	FMN-binding	0	SRR32559704
SARO_RS14845	Saro_2928	Sigma factor	0	SRR32559736

^*a*^ Peaks identified by macs3 are indicated along with links to the raw FASTQ data in NCBI SRA. Annotations are from RefSeq assembly GCF_000013325.1-RS_2019_12_16.

Sequencing reads for the protein-associated DNA libraries as well as the control library were trimmed to remove Illumina adapters using bbtools bbduk (v38.90, *k=21 mink=11 ktrim=r tbo tpe qtrim=r trimq=6 maq=10*) (10). The trimmed reads were aligned to the *N. aromaticivorans* DSM12444 genome (GCF_000013325.1, ASM1332v1) using Bowtie2 (v2.4.2, *--no-mixed --no-discordant*) (11). BigWig files were generated using deepTools (v3.5.0, --effectiveGenomeSize *4052498 --extendReads --binSize 1 --ignoreDuplicates --maxFragmentLength 600 --normalizeUsing RPGC*) (12). All control alignment files were merged using SAMtools (v1.1.1) (13). For each TF examined, peaks were identified using macs3 (v3.0.0a6, *--gsize 4052498 --call-summits --keep-dup 1*) (14). Of the 44 TFs selected for examination, 12 TFs lacked any identifiable peaks, while 32 TF samples had at least one peak identified and up to 1,205 peaks identified (Table 1). The DAP-seq data from these 32 TFs provide valuable insight into aromatic metabolism in *N. aromaticivorans*.

## Data Availability

All raw data are available at NCBI SRA under BioProject ID PRJNA1228656, and processed data are available at NCBI GEO under Accession GSE291618.
